# Histopathological features of the proper gastric glands in FVB/N-background mice carrying constitutively-active aryl-hydrocarbon receptor

**DOI:** 10.1186/s12876-019-1009-x

**Published:** 2019-06-21

**Authors:** Ai Dantsuka, Osamu Ichii, Annika Hanberg, Yaser Hosny Ali Elewa, Saori Otsuka-Kanazawa, Teppei Nakamura, Yasuhiro Kon

**Affiliations:** 1 Laboratory of Anatomy, Department of Basic Veterinary Sciences, Faculty of Veterinary Medicine, Hokkaido University, Sapporo, Japan, Kita 18, Nishi 9, Kita-ku, Sapporo, 060-0818 Japan; 2 Institute of Environmental Medicine, Karolinska Institutet, SE-171 77 Stockholm, Sweden; 3 Department of Histology and Cytology, Faculty of Veterinary Medicine, Zagazig University, Zagazig, 44519 Egypt; 4 Section of Biological Safety Research, Chitose Laboratory, Japan Food Research Laboratories, Bunkyo 2-3, Chitose, 066-0052 Japan

**Keywords:** Constitutively-active aryl-hydrocarbon receptor, Gastric mucosal abnormality, FVB/N mice, Cyst, Metaplasia

## Abstract

**Background:**

Aryl-hydrocarbon receptor (AhR) is a multiple ligand-activated transcription factor that has important roles in xenobiotic, physiological, or pathological functions. Transgenic mice systemically expressing constitutively-active AhR (CA-AhR) have been created to mimic activated AhR signaling in vivo. However, their detailed histopathological features are unclear. In the present study, we generated CA-AhR-expressing FVB/N mice (FVB-CA-AhR mice) and clarified their phenotypes in detail.

**Methods:**

Male and female FVB-CA-AhR and wild-type mice were histopathologically examined from 6 to 33 weeks of age.

**Results:**

Among the systemic organs, only the stomachs in FVB-CA-AhR mice showed pathological changes including cystic structures beneath the serosa; in addition, stomach weights increased with age. Histopathologically, cystic structures and alcian blue-positive metaplasia were observed in the mucosa of the proper gastric glands, and these two histometric parameters were positively correlated. Furthermore, proliferating cells shifted from the isthmus to the base of the glands, and parietal cells decreased. Age-related histopathological changes were clearer in females than in males. Importantly, in FVB-CA-AhR mice, intramucosal cysts developed as extramucosal cysts beneath the serosa, penetrating the lamina muscularis mucosae and the muscularis propria. Their incidence reached 100% in 28-week-old male mice and 33-week-old female mice. Extramucosal cysts contained alcian blue-, *Griffonia simplicifolia* lectin II-, or trefoil factor 2-positive cells, suggesting a stomach origin for the cysts and spasmolytic polypeptide-expressing metaplasia-like lesions.

**Conclusions:**

Disease onset occurred earlier in FVB-CA-AhR mice than previously reported in C57BL/6-derived CA-AhR mice. Importantly, the histopathological features were partly similar with gastritis cystica profunda in humans and animals. Excessive activation of AhR signaling aggravated abnormalities in the gastric mucosa and were affected by both genetic- and sex-related factors.

**Electronic supplementary material:**

The online version of this article (10.1186/s12876-019-1009-x) contains supplementary material, which is available to authorized users.

## Background

Aryl-hydrocarbon receptor (AhR) is widely expressed in various animal species and in humans. AhR is a multiple ligand-binding transcription factor and a member of the basic helix-loop-helix/Per-Arnt Sim domain proteins [1, 2]. AhR is constantly expressed in various organs and plays an important role in xenobiotic metabolism [3, 4]. Various ligands bind to AhR, followed by translocation into the nucleus and induction of the expressions of target genes, such as members of cytochrome P450 family, by binding to xenobiotic response elements in the promotor regions of these genes [5]. Environmental pollutants, including 2,3,7,8-tetracholorodibenzo-p-dioxin (TCDD), are well-known exogenous AhR ligands [3]. AhR also binds to endogenous ligands, including heme-, arachidonic acid-, or tryptophan-derived metabolites, such as 6-formylindolo[3,2-b]carbazole (FICZ) and indoxyl sulfate [6, 7].

Importantly, various AhR ligands affect the function and pathogenesis in digestive organs through feeding and metabolism. FICZ- and TCDD-induced AhR activation inhibits the development of mouse intestinal epithelial cells [8]. According to the aforementioned report, AhR was highly expressed in intestinal stem cells, and that FICZ affected the expression of downstream molecules of AhR signaling, such as Wnt family and bone morphogenetic proteins. Furthermore, AhR-null mice reportedly display increased incidence of inflammation-associated colorectal tumors caused by indole derivatives [9]. In the immune system, TCDD-induced activation of AhR signaling activates functional regulatory T-cells in mice [10]. Thus, the activation of AhR signaling may be involved in xenobiotic metabolism as well as the biological or pathological events associated with cell differentiation, proliferation, and inflammation [11, 12].

In humans, racial differences exist with respect to metabolic capacity [13] and incidence of diseases like gastric cancer [14]. These differences are crucial for diagnosis and medication. Since inbred strains of experimental rodents display different susceptibilities to diseases induced by bioactive substances, chemicals, and drugs, the appropriate selection of the genomic background of mouse models is crucial to investigations of the pathogenesis of different diseases in these models. For example, cerulein-induced pancreatitis is more severe in FVB/N mice than in BALB/c mice [15]. FVB/N mice also show unique phenotypes during the disease response. For instance, multidrug resistance 2 deficiency-induced hepatocarcinogenesis is significantly less severe in C57BL/6 mice than in FVB/N mice [16]. Although FVB/N mice are frequently used to generate transgenic mice [17], FVB/N-background transgenic mice carrying the *c-mos* oncogene [18] or mouse mammary tumor virus promoter-driven oncogenes [19, 20] harbor well-developed neoplasia compared with transgenic mice derived from other mouse strains [21, 22]. Thus, the FVB/N genomic background may exacerbate cell differentiation- and proliferation-related abnormalities.

MacGuire et al. developed a transgene encoding constitutively-active AhR (CA-AhR), which lacks a ligand-binding site (residues 288–421) in the Per-Arnt-Sim domain [23]. CA-AhR was utilized to mimic ligand-induced activation of AhR signaling. Transgenic mice expressing CA-AhR under the control of a modified simian virus 40 (SV40) promoter showed mucosal abnormalities in the glandular part of the stomach [24, 25]. In these studies, the stomach phenotypes were analyzed in mice derived from C57BL/6 or C57BL/6 × C3H hybrid mice.

In the present study, we generated FVB/N-background CA-AhR (FVB-CA-AhR) mice to examine the effects of different genomic backgrounds on CA-AhR-associated pathogenesis and to assess their pathogenic phenotypes in detail. The pathogenic phenotypes of FVB-CA-AhR mice differed in several aspects from those of CA-AhR mice created using another previously reported strain. These differences may be because of genetic factors associated with the FVB/N genomic background. Our data would be useful to understand pathogenesis induced by the activation of AhR signaling and its difference based on genomic diversity.

## Methods

### Animals

FVB-CA-AhR mice were created based on C57BL/6-background CA-AhR mice (B6-CA-AhR mice; created by Dr. Hanberg, Karolinska Institute, Stockholm, Sweden) at the National Institutes of Health (NIH; Bethesda, MD, USA) by backcrossing with FVB/N mice over six generations. FVB-CA-AhR mice used in the present study were kindly provided by Dr. Kopp (NIH) and were maintained along with FVB/N mice (CLEA Japan, Inc., Tokyo, Japan) in the Graduate School of Veterinary Medicine, Hokkaido University (Sapporo, Japan) under specific pathogen-free conditions. The gene encoding CA-AhR was genotyped using a PCR-based method with a primer pair (forward, 5′-TTACCTGGGCTTTCAGCAGT-3′; reverse, 5′-AACTGGGGTGGAAAGAATCC-3′), followed by agarose gel electrophoresis. FVB-CA-AhR mice were bred to homozygosity. Wild-type control mice included in this study were of the same mixed genetic background. All animals were handled in accordance with the Guide for the Care and Use of Laboratory Animals, Graduate School of Veterinary Medicine, Hokkaido University (approved by the Association for Assessment and Accreditation of Laboratory Animal Care International; experimental protocol approval no. 13–0032 and 16–0124).

### Sample collection

Male and female FVB-CA-AhR mice and the wild-type mice at 6–33-weeks (wks)-of-age were sacrificed by cervical dislocation under anesthesia (0.3 mg/kg medetomidine, 4.0 mg/kg midazolam and 5.0 mg/kg butorphanol) [26], and systemic organs were observed macroscopically. The stomach was removed from each mouse and kept in cold phosphate-buffered saline (PBS) to investigate histopathological features. After 5–10 min, stomach contents were removed by cutting the greater curvature and by rinsing with cold PBS. Each stomach was weighted, gently flattened, and pinned on a small piece of filter paper. Jejunum, pancrease, gallbladder, and liver were also collected from each mouse for lectin histochemistry. The collected organs were fixed with 4% paraformaldehyde, dehydrated with alcohol, and embedded in paraffin. To detect the proliferating cells, bromodeoxyulidine (BrdU, 100 mg/kg body weight) was intraperitoneally injected in some mice 2 h before euthanasia. For each age group, more than four mice were examined. Details are provided in Additional file 1: Table S1 and Additional file 2: Table S2.

### Histochemistry

Paraffin sections 3 μm in thickness containing non-glandular and glandular parts of the stomach that crossed the major axis along the lesser curvature were deparaffinized and hydrated. In addition to hematoxylin-eosin (HE) staining, periodic acid Schiff (PAS) or alcian blue pH 2.5 (AB pH 2.5) staining was performed to detect neutral mucins and acidic mucins, respectively.

Immunohistochemistry for cytochrome P450, family 1, subfamily a, polypeptide 1 (CYP1A1) was performed to determine stomach regions with CA-AhR expression-induced activation of AhR signaling. Furthermore, immunohistochemistry for proliferating cell nuclear antigen (PCNA) B220, CD3, pepsinogen, and caudal type homeobox 2 (CDX2) or trefoil factor 2 (TFF2) was done to evaluate the levels of proliferating cells, B-cells, pan T-cells, chief cells, and metaplasia, respectively. BrdU-incorporation was used to detect proliferating cells. Lectin histochemistry was performed by staining the stomach, jejunum, pancrease, gallbladder, and liver of individual mice with 21 types of biotinylated lectins (Vector Laboratories, Inc., Burlingame, CA, USA; Table 1). Details of primary and secondary antibodies, lectins, antigen retrievals, and blocking methods are summarized in Additional file 3: Table S3. Briefly, the paraffin sections were deparaffinized and antigens were retrieved for immunohistochemistry, but not for the lectin histochemistry. Next, slides were soaked in methanol containing 0.3% hydrogen peroxide (H_2_O_2_) to remove internal peroxidases. After washing with PBS, the sections were blocked with appropriate blocking reagents and were incubated overnight at 4 °C with primary antibodies or biotinylated lectins. Only for immunohistochemistry, after washing with PBS, sections were incubated with biotinylated secondary antibodies for 30 min at room temperature. Next, the sections were incubated with streptavidin–biotin complex (SABPO® kit; Nichirei, Tokyo, Japan) or goat anti-mouse IgG (SouthernBiotech, Birmingham, AL, USA) for 30 min. Colors were developed by incubating with 3, 3-diaminsobenzidine tetrahydrochloride-H_2_O_2_ solution. Finally, the sections were lightly counterstained with hematoxylin.

**Table 1:** Results of lectin histochemistory

Lectin name	target	FVB-CA-AhR	Wild-type								
		Extramucosal cyst	Stomach				Intestine		Liver	Gall-bladder	Pancreas
			Super-ficial	Neck	Parietal	Chief	Super-ficial	Goblet	Inter-lobular bile duct		inter-calated duct
Griffonia simplicifolia lectin II	GlcNAc a,b	+++	+	+++	±	++	–	–	–	–	–
Succinylated Wheat germ agglutinin	(GlcNAc)n	+++	+++	+++	±	–	–	±	–	±	+
*Ulex europaeus* aggultinin	Fuc a	++	++	–	+++	–	–	±	–	–	±
Griffonia simplicifolia lectin I	Gal a, GalNAc	++	++	–	++	–	–	±	–	++	±
Erythrina cristaggalli lectin	Gal b 1–4, GluNAc, Gal	++	++	+++	+	+++	+++	++	±	++	+
*Datura stramonium* lectin	(GlcNAc)n, Gal b1–4, GlcNAc	++	++	–	++	+	++	–	+	++	+
Wheat germ agglutinin	(GlcNAc)n, Sialic Acid	++	+	–	±	–	++	+	+++	++	±
Soybean agglutinin	GalNAc	++	+	–	++	–	+	+	+++	++	++
*Solanum tuberosum* lectin	(GlcNAc)n	+	+	+++	±	++	++	++	+	+++	+
*Lycopersicon esculentum* lectin	(GlcNAc)n	+	++	+++	±	–	++	±	++	++	+
*Vicia villosa* agglutinin	GalNAc	+	+++	++	++	–	+++	+	++	±	++
*Ricinus communis* agglutinin	Gal, GalNac	+	±	–	±	±	++	+	+	++	+
*Phaseolus vulgaris* leucoagglutinin	Gal/GalNAc−aOS^k^	+	–	–	+	+	++	+	++	–	+
Concanavalin A	Man α, Glc a	+	–	–	±	–	++	+++	±	++	–
*Arachis hypogaea* agglutinin	Gal b, 1–3 GalNAc	±	±	++	±	–	+++	++	++	+	+
Phaseolus vulgaris Erythroagglutinin	Gal/GalNAc−aOS^k^	±	±	+	±	±	+++	+++	+	+	+
*Sophora japonica* agglutinin	Gal b, GalNAc b	–	+++	+++	–	–	–	+	–	–	–
Dolochos biflorus agglutinin	GalNAc a	–	++	–	+++	–	++	±	+++	±	+++
*Pisum sativum* agglutinin	Man a	–	++	–	±	–	±	–	–	+++	±
Jacalin	Sialyl-Gal b 1–3, GalNAc-O-	–	–	–	±	++	+++	++	++	+	++
*Lens culinaris* agglutinin	Man a	–	±	–	±	–	±	–	–	+	+

For lectin histochemistry, the staining results were semi-quantitatively classified according to the intensity of positive staining reactions as follows: −, negative staining; ±, positive as well as negative staining; +, weak positive staining with light brown color; ++, positive staining with brown color; and +++, strong positive staining with dark brown color.

### Histoplanimetry

The proximal mucosal region of the stomach within 3 mm from the limiting ridge, which is the border between the non-glandular and glandular regions [24], was analyzed using three semi-serial sections a minimum interval of 30 μm. Rounded or oval expansion in the glandular lumen was defined as an “intramucosal cyst (IM cyst)”. The total number of IM cystss was determined in HE-stained sections. Cysts penetrating the muscularis propria were defined as “extramucosal cysts (EM cysts)”, and their incidence was calculated in all histologically examined mice.

The relative position of PCNA-positive (PCNA^+^) cells in the gastric mucosa was determined using ImageJ software (https://imagej.nih.gov/ij/) by calculating the area from the lamina muscularis to the limiting line showing the highest position of PCNA^+^ cells in each gastric pit divided by the area of the examined gastric mucosa. The calculated relative area (%) indicated the relative position of PCNA^+^ cells. The numbers of PCNA^+^ cells in the gastric mucosa and parietal cells in HE-stained sections, and the relative positive areas of PAS or AB pH 2.5, were analyzed using a model BZ-II Analyzer (Keyence, Osaka, Japan).

### Ultrastructure analysis

For scanning electron microscopy (SEM), the glandular part of the stomach besides the lesser curvature (approximately 1 cm^2^) was fixed with 2.5% glutaraldehyde for 2–4 h. After washing in 0.1 M phosphate buffer, the specimens were kept in 0.5% tannic acid for 10 min at 4 °C and in 1% for 1 h at 4 °C. The specimens were dehydrated through graded alcohol, transferred into 3-methylbutyl acetate, and dried using a model HCP-2 critical point dryer (Hitachi, Tokyo, Japan). The dried specimens were sputter-coated using a model E-1020 ion sputter coater (Hitachi), and examined using a model S-4100 microscope (Hitachi) with an acceleration voltage of 10 kV.

### Statistical analyses

All numerical results are presented as mean ± standard error (SE). Differences between groups were analyzed using a non-parametric method (Mann–Whitney *U* test). Correlation between the number of EM cysts and AB pH 2.5^+^ areas was analyzed using Spearman’s correlation coefficient test (ρ). In the analyses, significance was indicated by *P* < 0.05.

## Results

### Systemic phenotypes of FVB-CA-AhR mice

**Fig. 1:**
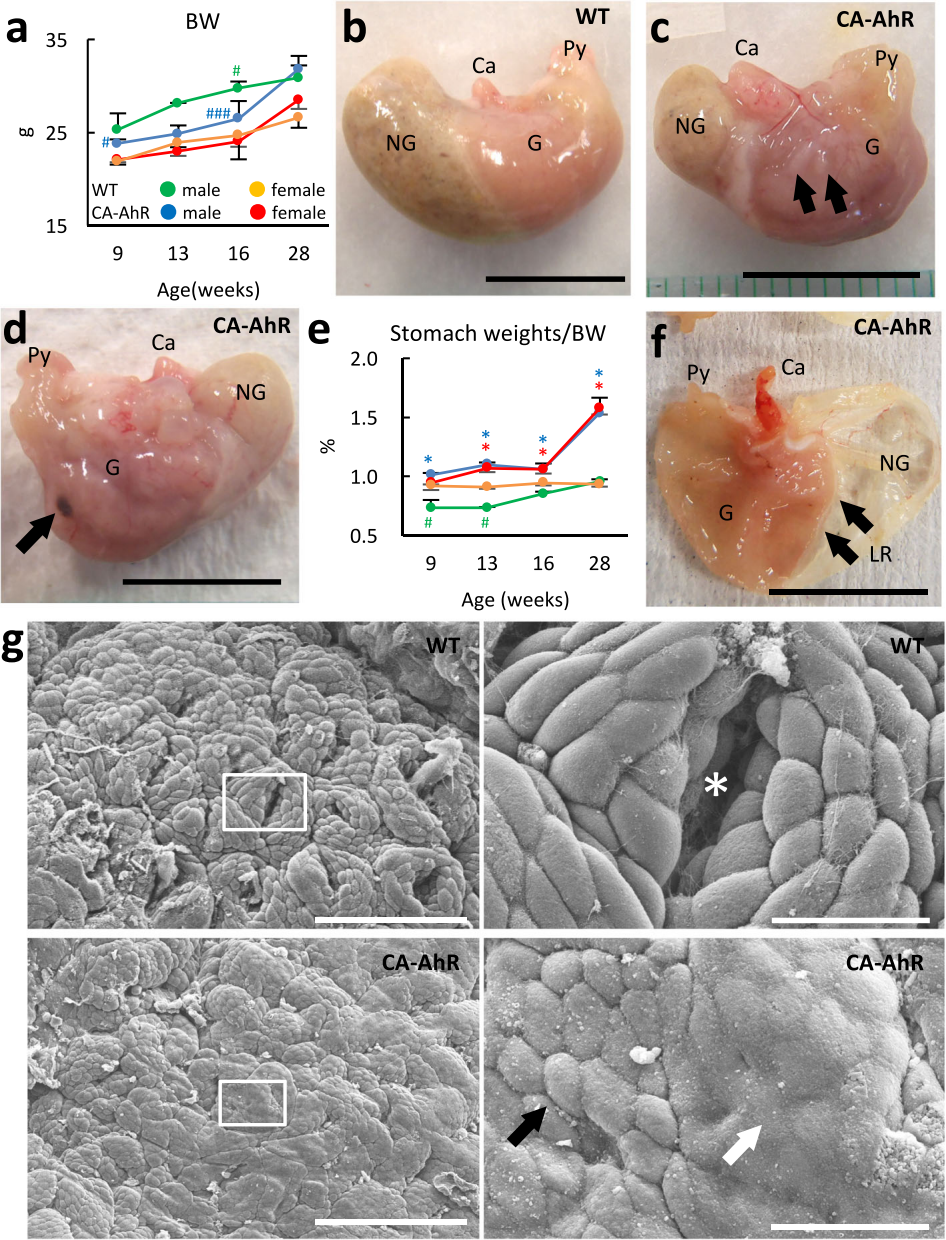
Body weight, stomach weight, and macroscopic features of stomachs in FVB-CA-AhR and wild-type mice. (**a**) Body weight of mice. (**b**-**d**) Representative macroscopic features of the mouse stomach. b: No lesions were evident in a 28 wk. wild-type (WT) male. **c**: Cystic structures were evident (arrows) in a 9 wk. FVB-CA-AhR (CA-AhR) male. d: Hemorrhagic lesion (arrow) in a In 28 wk. CA-AhR male. Abbreviations: Ca: cardia, G: glandular part, NG: non-glandular part, and Py: pylorus. (**e**) Relative weight of the stomach to body weight. (**f**) Representative macroscopic features of the gastric mucosa on a 28 wk. female. Arrows denotes the limiting ridge (LR). (**g**) Ultrastructure of the gastric mucosa revealed by scanning electron microscopy. Asterisk: gastric pit, black arrow: mucous secreting cell, and white arrow: fused mucous secreting cells. CA-AhR denotes FVB-CA-AhR. In the graphs, the values are expressed as mean ± SE. Blue and red asterisks denote significant strain differences with wild-type (WT) mice in males and females at the same age, respectively (Mann-Whitney-*U* test*, P* < 0.05). Sharp denotes significant sex-related difference in the same strains at the same ages (Mann-Whitney-*U* test*,* ###*P* < 0.001, #*P* < 0.05). Bars denote 1 cm (**b**–**d**), 500 μm (f), 100 μm (**g**, left panels), and 20 μm (right panels)

No FVB-CA-AhR mice died during the observation period (6–33 wks). The mice were fertile and produced an equal sex ratio in pups. Body weights of FVB-CA-AhR and wild-type mice increased comparably with age, and significant sex-related differences were observed in both FVB-CA-AhR (at 9 and 16 wks) and wild-type mice (at 16 wks) (Fig. 1a). Macroscopic observation of systemic organs revealed severe pathological changes only in the stomachs of FVB-CA-AhR mice. No macroscopic changes were observed in the stomachs of wild-type mice (Fig. 1b). In contrast, stomachs of FVB-CA-AhR mice showed cystic structures beneath the serosa on the parietal and visceral surfaces (Fig. 1c); moreover, these structures were only localized around the lesser curvature. These cystic structures appeared from 8 wks in males and 10 wks in females, and increased in size and number with age (Fig. 1d). Hemorrhagic lesions were observed in a few aged mice (Fig. 1d). Relative stomach weight to body weight was significantly increased in FVB-CA-AhR mice compared with wild-type mice. However, significant sex-related differences were observed only in wild-type mice at 9 and 13 wks (Fig. 1e). Although macroscopic examination showed normal gastric mucosa in all the examined mice (Fig. 1f), microscopic examination of the mucous surface yielded different results for FVB-CA-AhR and wild-type mice. In wild-type mice, each gastric pit was clearly observed, and the border between each mucous secreting cell was clear and well-organized (Fig. 1g). In contrast, FVB-CA-AhR mice showed obscure gastric pits and cell borders, with partially fused surfaces of some cells (Fig. 1 g).

### Histopathological features of stomachs in FVB-CA-AhR mice

**Fig. 2:**
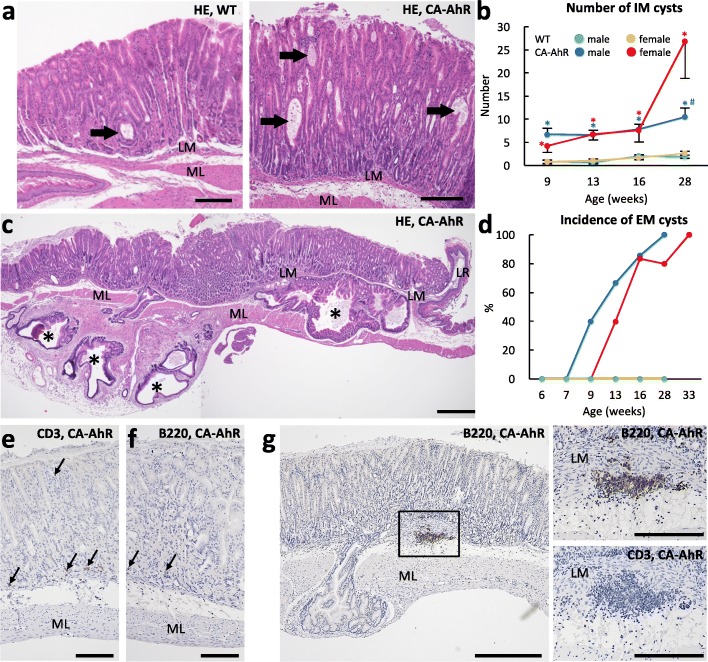
Intramucosal cyst and cell infiltration in gastric mucosa of FVB-CA-AhR and wild-type mice. (**a**) Proper gastric glands in 9 wk. males revealed by HE staining of sections. Arrows denote intramucosal (IM) cysts. (**b**) The number of IM cysts. For graphs, blue and red asterisks denote significant strain difference with wild-type (WT) mice in males and females at same ages, respectively (Mann-Whitney-*U* test*, P* < 0.05). Sharp denotes significant sex-related difference in the same strains at the same age (Mann-Whitney-*U* test*,* #*P* < 0.05). (**c**) Proper gastric glands in 28 wk. males revealed by HE staining of sections. Asterisks denote extramucosal (EM) cysts. (**d**) Incidence of EM cysts. Values are expressed as percentage. (**e**–**g**) B220^+^ and CD3^+^cells in the proper gastric glands revealed by immunohistochemistry. Arrows denote positive cells; **e** and **f**: 6 wk. male, **g**: 16 wk. male. CA-AhR denotes FVB-CA-AhR. Abbreviations are: LM: lamina muscularis, ML: muscle layer, and LR: limiting ridge. Bars denote 200 μm (**a**), 500 μm (**c**), 100 μm (**e** and **f**), 500 μm (**g**, left panel), and 20 μm (**g**, right panels)

Wild-type mice showed IM cysts in different regions of the proper gastric glands. These cysts were lined with simple cuboidal or columnar epithelial cells (Fig. 2a). In contrast, FVB-CA-AhR mice showed numerous IM cysts (Fig. 2a). The number of IM cysts was significantly higher in FVB-CA-AhR mice than in wild-type mice, with a slight increase in cysts with age (Fig. 2b). The number of IM cysts was significantly higher in female FVB-CA-AhR mice than in male mice at 28 wks (Fig. 2b). Only in FVB-CA-AhR mice, some IM cysts were connected to EM cysts through the lamina muscularis as well as muscularis propria (Fig. 2c). The histopathological structure of EM cysts became complicated with branching, which increased with age (Fig. 2c). However, no sex-related histopathological differences were observed in EM cysts. EM cysts first appeared from 8 wks of age in males and from 10 wks of age in females, and their appearance corresponded with the appearance of macroscopically evident cystic structures (Fig. 1). The incidence of EM cysts increased with age in both sexes of FVB-CA-AhR mice (Fig. 2d). Infiltrations of B220^+^ B-cells or CD3^+^T-cells in the gastric mucosa were not severe during the observation period (Fig. 2e and f). In some FVB-CA-AhR mice with EM cysts, B220^+^ B-cells infiltrated beneath the lamina muscularis (Fig. 2g). However, there were few B220^+^ B-cells around EM cysts in younger mice.

**Fig. 3:**
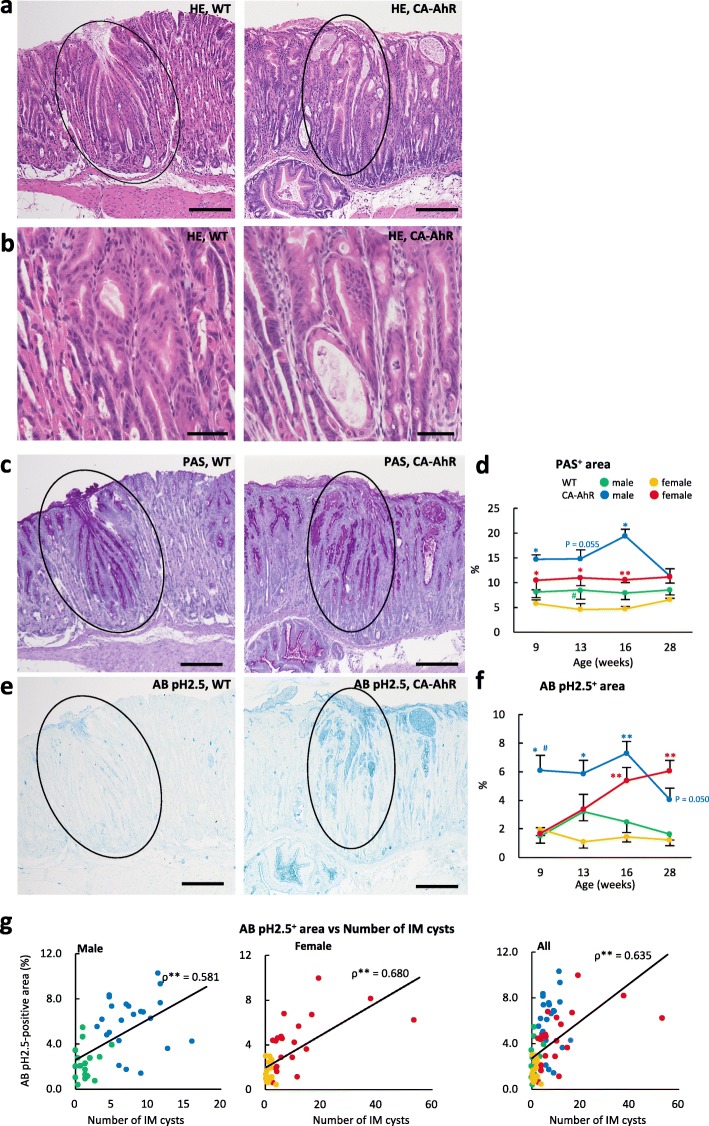
Metaplasia in gastric mucosa of FVB-CA-AhR and wild-type mice. (**a** and **b**) Proper gastric glands in 16 wk. males revealed by HE staining. (**c**) PAS^+^ area in the proper gastric glands in 16 wk. males revealed by PAS staining. (**d**) Relative PAS^+^ area in the proper gastric glands. (**e**) AB pH 2.5^+^ area in proper gastric glands in 16 wk. males revealed by AB pH 2.5 staining. (**f**) Relative AB pH 2.5^+^ area in the proper gastric glands. (**g**) Correlation between IM cysts and intestinal metaplasia. Graph shows the Spearman’s rank correlations between the number of IM cysts and relative AB pH -2.5^+^ area in the proper gastric glands. *P < 0.05, Spearman’s rank correlation test; ρ Spearman’s rank correlation coefficient; CA-AhR: FVB-CA-AhR. In **a**, **d**, and **e**, the circles denote metaplastic areas in serial sections. In graphs, blue and red asterisks denote significant strain difference with wild-type (WT) mice in male and female at the same age, respectively (Mann-Whitney-*U* test*,* *P < 0.05, **P < 0.01). Sharp denote significant sex-related difference in the same strains at the same age (Mann-Whitney-*U* test*,* #*P* < 0.05). Bars denote 200 μm (**a**, **c**, and **e**) and 50 μm (**b**)

The morphology of epithelial cells in some regions of the proper gastric glands changed to a columnar shape in FVB-CA-AhR and wild-type mice, indicating epithelial metaplasia (Fig. 3a and b). PAS^+^ substances were localized in the superficial regions of the normal mucosa in wild-type mice, and were enlarged toward the basal region of metaplastic lesions in both wild-type and FVB-CA-AhR mice (Fig. 3c). The relative PAS^+^ area was significantly greater in FVB-CA-AhR than in wild-type mice, and no age-related changes were observed in any of the examined mice (Fig. 3d). Male FVB-CA-AhR and wild-type mice showed higher values than the female counterpart mice. However, a significant sex-related difference was observed only in wild-type mice at 13 wks.

A previous report [27] described metaplastic lesions that were positive for PAS and AB pH 2.5. Presently, in the wild-type mice, the PAS^+^ area was negative for AB pH 2.5 staining (Fig. 3e). In contrast, some PAS^+^ areas in FVB-CA-AhR mice displayed AB pH 2.5 positivity, which was pronounced in metaplastic lesions (Fig. 3e). Histoplanimetry revealed that the relative AB pH 2.5^+^ area was significantly greater in FVB-CA-AhR mice than in wild-type mice (Fig. 3f). Characteristically, the relative AB pH 2.5^+^ area was higher in male FVB-CA-AhR mice than in wild-type mice from 9 wks of age, but that in female FVB-CA-AhR mice increased with aging. Moreover, a significant correlation was observed between the number of IM cysts and AB pH 2.5^+^ area in males, females, and all examined mice (Fig. 3g).

### Proliferative features of altered cells in gastric mucosa of FVB-CA-AhR mice

**Fig. 4:**
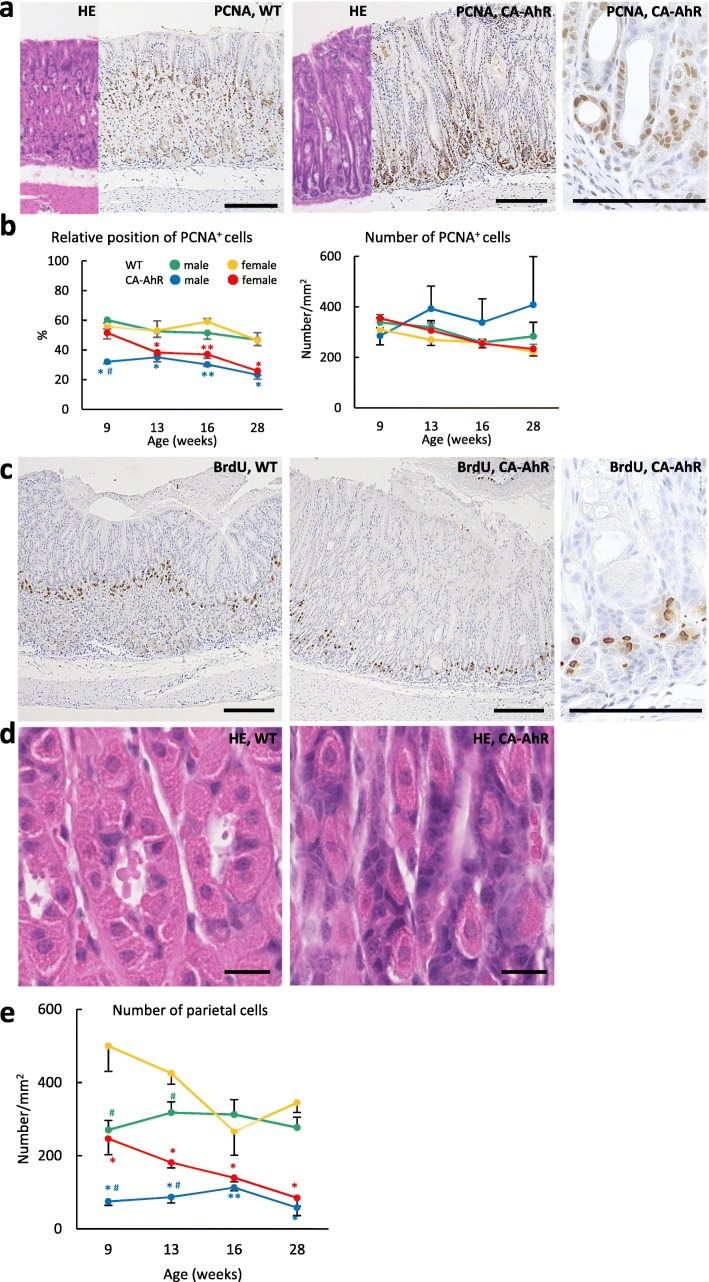
Proliferating cells and parietal cells in proper gastric glands of FVB-CA-AhR and wild-type mice. (**a**) PCNA^+^ cells in the proper gastric gland of 9 wk. males revealed by HE staining (left panel) and immunohistochemistry (right panel). (**b**) Relative position and the number of PCNA^+^ cells in the proper gastric gland. (**c**) BrdU-incorporated cells in the proper gastric gland of 16 wk. males revealed by . immunohistochemistry. (**d**) Body in the proper gastric gland of 9 wk. males revealed by HE staining. (**e**) The number of parietal cells in the proper gastric gland. CA-AhR: FVB-CA-AhR. In graphs, blue and red asterisks denote significant strain difference with wild-type (WT) mice in male and female at the same age, respectively (Mann-Whitney-*U* test*,* *P < 0.05, **P < 0.01). Sharp denotes significant sex-related difference in the same strains at the same age (Mann-Whitney-*U* test*,* #: *P* < 0.05). Bars denote 200 μm (**a** and **c**) and 20 μm (**d**)

Nuclear PCNA positivity was observed from the isthmus to the neck region in proper gastric glands in wild-type mice, and from the body to the base of the glands in FVB-CA-AhR mice (Fig. 4a). Histoplanimetry revealed that the relative positions of PCNA^+^ cells decreased with age in all the examined mice, and were significantly lower in FVB-CA-AhR mice than in wild-type mice (Fig. 4b), indicating a downward shift in the cell proliferation zone. A sex-related difference was observed only in FVB-CA-AhR at 9 wks, with male mice displaying lower values than female mice. However, the number of PCNA^+^ cells in the gastric mucosa was comparable between wild-type and FVB-CA-AhR mice (Fig. 4b). Altered positions of PCNA^+^ cells in FVB-CA-AhR mice were also confirmed by the detection of cells that incorporated BrdU (Fig. 4c). Moreover, the parietal/chief cell zone, which normally occupies the region of mucous two-thirds from the bottom, decreased with a change in the position of the cell proliferation zone (Fig. 4a and d). The number of parietal cells was significantly lower in FVB-CA-AhR mice than in wild-type male mice during the examination period, and the cell number in female mice was significantly decreased with age; however, this was not observed in wild-type mice (Fig. 4e). Significant sex-related difference was detected in FVB-CA-AhR and wild-type mice at 9 and 13 wks, and males displayed lower values than females (Fig. 4e).

### Histopathological characteristics of EM cysts in FVB-CA-AhR mice

**Fig. 5:**
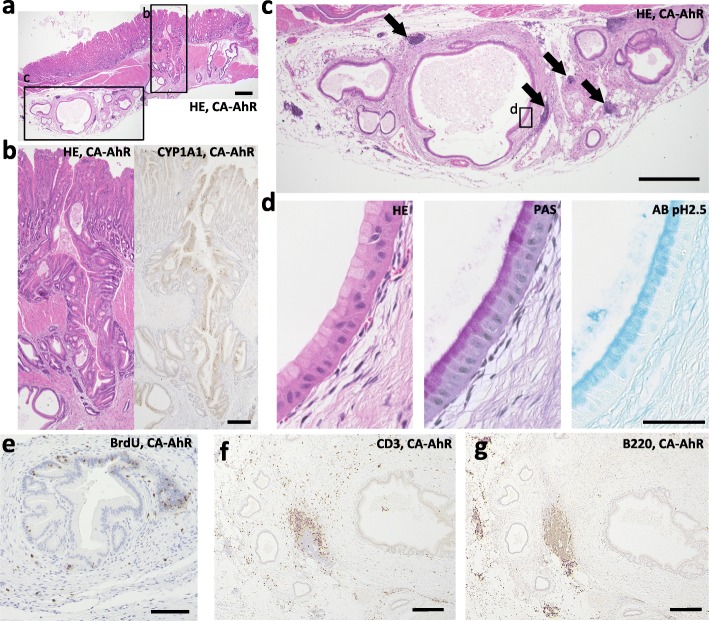
Histological characteristics of EM cysts in FVB-CA-AhR mice. (**a**) Proper gastric gland in 28 wk. male revealed by HE staining. (**b**) Squared area in panel a presents HE staining (left panel) and immunohistochemistry for CYP1A1 (right panel). (**c**) Squared area in panel **c** presents HE staining. Arrows: lymphoid clusters. (**d**) Squared area in panel **d**. HE, PAS, and AB pH 2.5 staining. (**e**–**g**) Positive cells determined by immunohistochemistry for BrdU (**e**), CD3 (**f**), and B220 (**g**) in or around the EM cysts in a 16 wk. male. CA-AhR: FVB-CA-AhR. Bars denote 500 μm (**a** and **c**), 200 μm (**b**, **e**-**g**), and 50 μm (**d**)

We histopathologically examined the EM cysts in aged FVB-CA-AhR mice in detail (Fig. 5). Some EM cysts contained cell debris and seemed to continue from the entrance of gastric pit (Fig. 5a and b). Positive reaction for CYP1A1, indicating the activation of the AhR signaling pathway, was detected in the lining epithelium from the gastric pits to the penetrating EM cysts (Fig. 5b). Some lymphoid clusters were observed around EM cysts (Fig. 5c). Epithelial cells lining the EM cysts were morphologically similar to mucous secreting cells in the proper gastric glands. These cells were columnar shaped (Fig. 5d) and showed both PAS^+^ and AB pH 2.5^+^ reactions (Fig. 5d). Some cells incorporated BrdU (Fig. 5e), indicating the existence of proliferating cells. Fat tissues, blood vessels, and mild CD3^+^T-cell infiltrations were observed around EM cysts, but there were few B220^+^ B-cells (Fig. 5f and g). Lymphoid clusters around EM cysts contained both CD3^+^ T-cells and B220^+^ B-cells (Fig. 5f and g). In contrast, no other cell type, such as parietal cells and chief cells, which are present in the proper gastric glands were observed. No dysplastic features were observed.

**Fig. 6:**
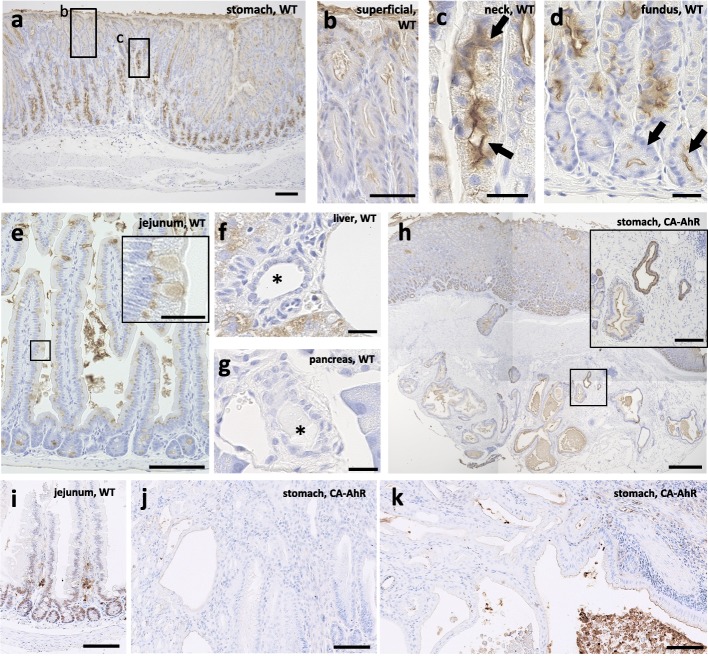
Localization of GSII and CDX2 in digestive organs of FVB-CA-AhR and wild-type mice. (**a**-**h**) Lectin histochemistry for GSII and (i-k) immunohistochemistry for CDX2. (**a**) Proper gastric gland of a 16 wk. male examined by lectin histochemistry. (**b**) Squared area in panel **a**. (**c**) Squared area in panel a. Arrows denote GSII^+^ mucous neck cells. (**d**) The base of the glands in the proper gastric gland in another 16 wk. male. Arrows denote GSII^+^ chief cells. (**e**) The jejunum of a 16 wk. male examined by lectin histochemistry. Inset shows goblet cells. (**f**) The liver of a 16 wk. male examined by lectin histochemistry. Asterisk denotes interlobular bile duct. (**g**) The pancreas of a 16 wk. male examined by lectin histochemistry. Asterisk denotes intercalated duct. (**h**) The proper gastric gland of a 28 wk. male examined by lectin histochemistry. Inset displays EM cysts. (**i**) The jejunum of a 33 wk. female. (**j**) The proper gastric gland including IM cysts in a 33 wk. female. (**k**) EM cysts in a 33 wk. female. CA-AhR: FVB-CA-AhR; WT: wild-type; Bars denote 500 μm (**a** and **h**), 100 μm (**e**, **i**-**k**), 50 μm (**b**), and 20 μm (**c**, **d**, **f**, **g**, and insets)

To investigate the characteristics of EM cysts, especially their epithelial features, histochemical analysis was done using 21 types of lectins. The staining patterns were compared to the mucous in the healthy stomach as well as in other organs, including the intestine (jejunum), liver (interlobular bile duct), gallbladder, and pancreas (intercalated duct). The results are summarized in Table 1. Figure 6 shows the results of staining with a representative lectin *Griffonia simplicifolia* lectin II (GSII), which produced strong staining of epithelial cells in the stomach (Table 1). GSII^+^ staining was detected in superficial, neck, parietal, and chief cells in wild-type mice (Fig. 6a–d), with neck cells showing strong staining (Fig. 6c). However, GSII^+^ staining was not detected in the other organs (Table 1 and Fig. 6e–g). In the EM cysts, GSII^+^ staining was clearly observed in lining cells, which morphologically resemble mucous neck cells and which do not secrete much mucous (Fig. 6h). The collective lectin staining patterns in EM cysts closely matched the pattern of glandular cells of the stomach, nearly matched the pattern in the intestine, and did not match patterns in other organs (Table 1).

To clarify the characteristic of metaplasia found in the stomach of FVB-CA-AhR mice at 33 wks, we performed immunohistochemistry for CDX2 (Fig. 6i-k), a marker for intestinal metaplasia. Lesions in FVB-CA-AhR mice were negative for CDX2 but a positive reaction was obtained in the jejunum (Fig. 6i-k).

**Fig. 7:**
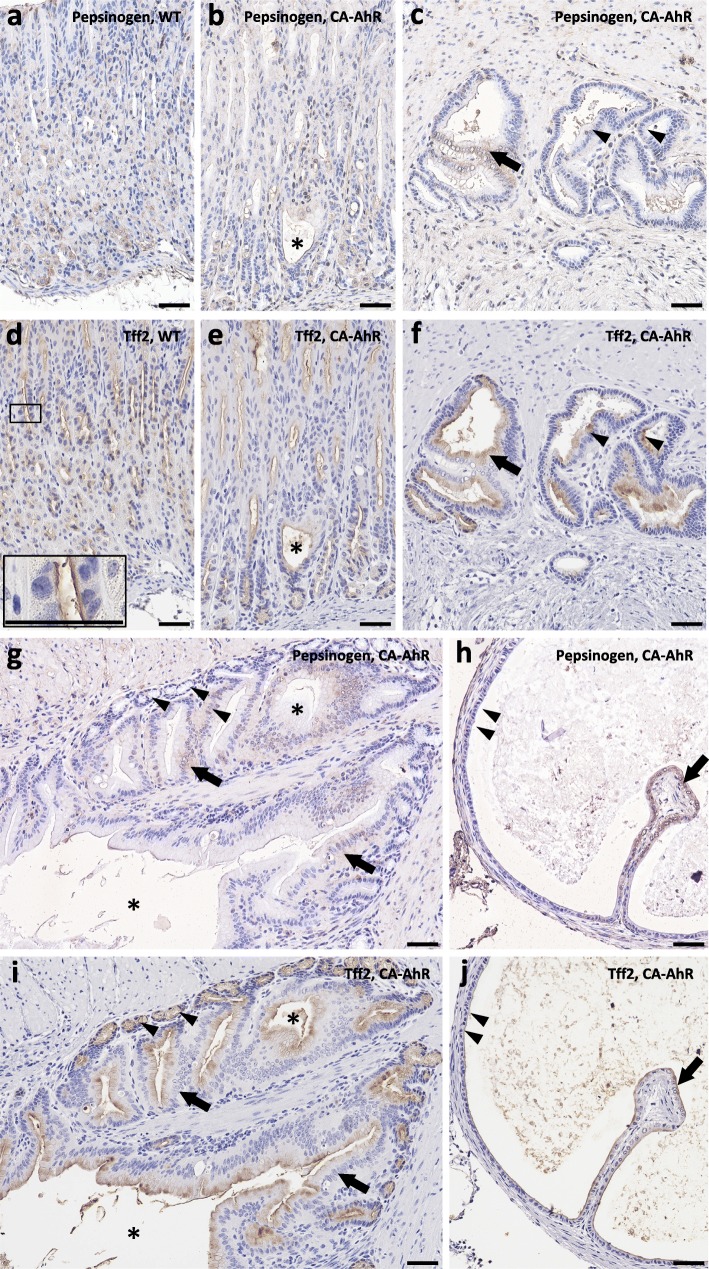
Localization of pepsinogen and TFF2 in stomachs of FVB-CA-AhR and wild-type mice. (**a**-**c**, **g** and **h**) Immunohistochemistry for pepsinogen. (**d**-**f**, **i** and **j**) Immunohistochemistry for TFF2. (**a** and **b**) The proper gastric gland in a 33 wk. female. Asterisk denote an IM cyst. (c) EM cysts in a 33 wk. female. Arrow denotes pepsinogen^+^ cells and arrowheads denote pepsinogen^−^ cells. (**d** and **e**) Serial sections to panels **a** and **b**. Insets: TFF2^+^ cells; asterisk: IM cyst. (**f**) Serial section to panel **c**. Arrow: TFF2^+^ cells; arrowheads: TFF2^−^ cells. (**g** and **h**) Well-developed EM cysts in a 33 wk. female. Arrows denote pepsinogen^+^ cells and arrowheads denote pepsinogen^−^ cells. (**i** and **j**) Serial sections to panels **g** and **h**. Arrow denotes TFF2^+^ cells and arrowheads denote TFF2^−^ cells. CA-AhR: FVB-CA-AhR; WT: wild-type; Bars denote 50 μm

Immunohistochemistry of serial sections examined pepsinogen and TFF2 to examine the development of spasmolytic polypeptide-expressing metaplasia (SPEM)-like lesions in FVB-CA-AhR mice at 33 wks (Fig. 7). The positive reactions for pepsinogen tended to be weak in FVB-CA-AhR mice compared to the wild-type mice (Fig. 7a and b). Furthermore, FVB-CA-AhR mice showed weakly positive pepsinogen reactions in cells in the apical portion of IM cysts (Fig. 7b). In EM cysts, pepsinogen negative and positive cells were observed (Fig. 7c). TFF2 positive reactions were mainly observed in cells located in the apical region of the neck in wild-type mice. There were more TFF2 positive cells in CA-AhR mice (Fig. 7d and e). In CA-AhR mice, most IM and EM cysts were positive for TFF2 (Fig. 7e and f). In well-developed EM cysts, very few cells showed positive reactions for pepsinogen (Fig. 7g and h). TFF2^+^ reactions were mainly observed at the apical portion of large EM cyst lining cells (Fig. 7i) and some epithelial cells were positive for both pepsinogen and TFF2 (Fig. 7j).

## Discussion

In FVB-CA-AhR mice, the pathological phenotypes characterized by cyst formation and epithelial metaplasia in the mucosa were observed only in the stomach, even though CA-AhR expression was driven by the modified SV40 systemic promoter [25]. AhR activation is crucial for xenobiotic metabolism and excessive AhR activation alters the severity of several diseases in the digestive system, including the liver and intestinal tract [28–31]. Although AhR is widely expressed in systemic organs, our results suggest that the stomach is more sensitive to excessive AhR activation. Previous studies have reported that B6-CA-AhR mice develop stomach-specific lesions [24, 25]. Therefore, cell-specific function of AhR, different expression levels of CA-AhR in different organs, or localization patterns of AhR partner molecules might be important for the development of organ-specific phenotypes in CA-AhR mice, while the difference in genomic backgrounds are not important.

Formation of numerous IM cysts and AB pH 2.5^+^ epithelial metaplasia in the proper gastric glands were characteristic features in FVB-CA-AhR mice. The present study clarified the histological features of IM cysts in CA-AhR mice in detail. Similar cystic lesions in the gastric mucosa have been reported in gastric hyperplastic polyps and in patients receiving long-term proton pump inhibitor therapy [32, 33]. In the present study, these lesions were also observed in wild-type mice, and the number of IM cysts was slightly increasing with age. However, the pathological mechanisms underlying the development of these lesions are unknown. Importantly, a correlation was observed between histoplanimetrical parameters of IM cysts and metaplasia, suggesting their association with pathological events occurring in the gastric mucosa. However, the causal relationship underlying this association is unclear. Furthermore, in FVB-CA-AhR mice, the number of cell proliferation features and inflammation severity in the gastric mucosa did not change with an increase in the number of IM cysts and metaplasia. These findings suggest that excessive activation of the AhR signaling pathway exacerbates age-related stomach lesions without promoting the formation of new lesions associated with cell proliferation or inflammation. Metaplasia and EM cysts did not prelude lethal lesions, such as carcinogenic lesions, during the examination period.

The most notable feature of the stomachs in FVB-CA-AhR mice was the formation of EM cysts. Interestingly, we detected a connection between IM and EM cysts, i.e., BrdU-incorporated cells, and the mucin secreting cells lining the EM cysts. These data suggest that IM cysts elongate to form EM cysts that feature cell proliferation and luminal secretion. Furthermore, the structures of EM cysts became complex and branched in aged FVB-CA-AhR mice. Therefore, we assumed that abnormal and ectopic development of ductal structures, such as pancreatic or bile ducts, which has been reported in humans [34, 35], was associated with the formation of EM cysts. However, histochemical analysis with lectin revealed a similar glycosylation pattern between epithelial cells lining the EM cysts and epithelial cells present in the proper gastric glands, and dissimilarity between epithelial cells lining the EM cysts and cells in the pancreas or liver. Furthermore, the immunohistochemistry analysis revealed that the lesions in FVB-CA-AhR mice were similar to TFF2^+^ SPEM rather than CDX2^+^ intestinal metaplasia. Recent studies indicated that loss of parietal cells due to acute toxic injury or chronic infection by *Helicobacter pylori* cause the transdifferentiation of chief cells into SPEM metaplastic cells [36]. Furthermore, in SPEM the decreased expression of chief cell markers, such as basic helix-loop-helix family member a15 (also known as MIST1), and increased expression of TFF2 was observed in the metaplasia lesion [37]. We also clarified the strong and weak expression of TFF2 and pepsinogen, respectively, in cells lining EM cysts in FVB-CA-AhR mice. These data suggest the association of CA-AhR with the abnormal differentiation of chief cells. However, there has been no report associating SPEM with the activation of AhR.

The decrease in the number of parietal cells found in FVB-CA-AhR mice would be a key factor in the pathogenesis of cyst formation in the stomach. Mice lacking the *Atp4a* gene, whose product has crucial functions in parietal cells, develop IM cysts [38]. EM cysts can also develop with the loss of parietal cells [39–42]. Furthermore, loss of function of parietal cells induces epithelial metaplasia [41]. However, the present study as well as previous studies could not show a direct association between AhR activation and parietal cell abnormalities. In contrast, the downward shift of the cell proliferation zone from the isthmus to the neck region in FVB-CA-AhR mice suggests AhR activation-induced abnormal differentiation of cells in the gastric pits. Similar histological features were reported for mice with a *Kras*^G12D^ mutation [43]. However, the relationship between the decrease in the number of parietal cells and the downward shift of the cell proliferation zone is unclear because these events occurred simultaneously in the present and a previous study. A recent study reported that Lgr5^+^ stem cells express AhR, and that AhR ligands inhibit the in vitro development of mouse intestinal organoids [8]. Localization of Lgr5^+^ stem cells has also been described in the stomachs of mice [44] and humans [45]. AhR can influence apoptotic or anti-apoptotic effects, participate in growth factor signaling, and regulate the cell cycle [46]. Therefore, AhR activation might directly or indirectly affect the cell differentiation process in the gastric mucosa, including parietal cells, and lead to the formation of cystic and metaplastic lesions. Further studies to clarify the functional crosstalk between AhR activation and Lgr5^+^ stem cells are needed.

Histopathological features of stomach lesions were similar between FVB-CA-AhR and B6-CA-AhR mice (examined in a previous study, Table 2). However, the onset of disease symptoms, such as EM cyst development, occurred earlier in FVB-CA-AhR mice (8 and 10 wks in male and female mice, respectively) than in B6-CA-AhR mice (10 and 17 wks in male and female mice, respectively) [24]. Furthermore, the incidence of EM cysts reaching 100% occurred earlier in FVB-CA-AhR mice (28 and 33 wks in male and female mice, respectively) than in B6-CA-AhR mice (52 wks and after 52 wks in male and female mice, respectively) [24]. These strain-related differences may reflect the difference in the genomic backgrounds between the two mouse strains. Particularly, mice with the FVB/N genomic background might have adverse abnormalities of cell differentiation and proliferation [47], FVB/N-derived genomic factors promote AhR activation-induced phenotype development in the gastric mucosa compared with B6-derived genomic factors.

**Table 2:** Summary of differences between FVB-CA-AhR and B6-CA-AhR mice

	FVB-CA-AhR		B6-CA-AhR [24]	
	Male	Female	Male	Female
EM cyst development	8 wks	10 wks	10 wks	17 wks
Incidence of EM cysts reached 100%	28 wks	33 wks	52 wks	52 wks
CDX2-positivety	–	–	NE	NE
TFF2-positivity	Strong	Strong	NE	NE
Pepsinogen-positivity	Weak	Weak	NE	NE

*wks* weeks of age, *NE* not examined

Several sex-related differences in phenotypes were observed in FVB-CA-AhR mice. Particularly, the number of IM cysts significantly increased with age in females than in males, and the development of EM cysts occurred earlier in males than in females. These differences might be caused by sex hormones or by mucosal stimulation due to increased food intake in male mice. In humans and experimental animals, estrogen exerts protective effects in the stomach and plays an important role in reducing the risk of gastric cancer [48, 49] or in attenuating pathological changes in the gastric mucosa [50, 51]. 9,10-Dimethyl-1,2-benzanthracene-activated AhR signaling induces estradiol production by regulating the expression of *Cyp19* [52]. In addition, crosstalk exists between AhR and estrogen receptors (ERs). AhR induces the expression of genes encoding CYP1 family of estrogen-metabolizing enzymes, initiates the degradation of ERs, and suppresses estrogen signaling pathways. In contrast, ERs function as AhR coactivators to modulate the expression of *CYP1A1* and *CYP1B1* [53]. These findings suggest that sex hormones, especially estrogen, modify CA-AhR-induced phenotypes in the stomach. These phenotypes may be exacerbated in aged female FVB-CA-AhR mice because of the unbalanced production of sex hormones.

Histopathological characteristics of the stomachs of FVB-CA-AhR mice were similar to those of the stomachs of humans and animals with gastritis cystica profunda (GCP). In humans, GCP are occasionally found along with polypectomy [54] and remnant cancers [55], and also develop in non-operated stomach. Cells lining GCP are mainly single-layered mucous secreting cells showing both PAS^+^ and AB pH 2.5^+^ reactions, and come cells incorporate BrdU [56], similar to those present in the EM cysts in FVB-CA-AhR mice. Some have researchers argued that GCP is a precancerous lesions because multiple cancers frequently develop with GCP [57, 58]. However, this view is debatable. Several studies have reported GCP development in experimental animals, such as mice or Mongolian gerbils, infected with *Helicobacter spp.* [40, 59] and in mice lacking the *Kcne2* gene [41], and amphiregulin [60]. Amphiregulin, an epidermal growth factor receptor ligand secreted by parietal cells, is an AhR ligand, and the expression of the gene encoding amphiregulin is induced by TCDD [61, 62]. Importantly, Roepke et al. reported that that *Kcne2* knockout mice that displayed parietal cell dysfunction, exhibit GCP characterized by increased stomach mass, increased Ki67 expression, and TFF2-expressing metaplasia [41]. This data support three of our findings in particular—that proliferating cells shift from the isthmus to the base of glands, parietal cells decrease, and SPEM-like lesions form in CA-AhR mice. Determination of the relationships between epithelial growth factors and AhR in the stomach, particularly in parietal cells, will increase our understanding of the mechanisms underlying GCP development. CA-AhR mice might be useful for determining these relationships.

Direct evidence of an association between AhR activation and SPEM or GCP in humans has not been obtained. An immunohistochemical study reported gradually increasing levels of AhR expression, with the lowest levels in superficial gastritis, followed by chronic atrophic gastritis, intestinal metaplasia, atypical hyperplasia, and gastric cancer. Furthermore, AhR expression and nuclear translocation were significantly increased in a severe phenotype, such as gastric cancer [63]. Interestingly, coffee can activate the AhR pathway [64], indicating the relationship between human food consumption and AhR in the stomach. Furthermore, the stomach expresses cytochrome P450 isoforms including CYP1A1 [65] and significant associations between CYP1A1 polymorphisms and gastric cancer risk have been reported in humans, suggesting that the altered function of AhR-downstream molecule induces the stomach abnormality [66]. These data emphasize the pathological crosstalk between the stomach and the AhR pathway in humans.

## Conclusions

FVB-CA-AhR mice developed invasive cystic glands that penetrated the lamina muscularis as well as muscularis propria. Several sex-related differences were evident. These glands developed earlier than previously described CA-AhR mice. Before the development of the cystic glands, the epithelium of the gastric mucosa showed aggravated cyst formation and TFF2-positive SPEM-like lesions, and the cell proliferation zone shifted from the isthmus to the base of the glands. These histopathological features are partly similar to GCP in humans and animals. These results suggest that excessive activation of the AhR signaling pathway aggravates mucosal abnormalities in the stomach epithelium, and that these pathological events are affected by both genetic- and sex-related factors.

## Additional files

Additional file 1: **Table S1.** Number of examined mice in Figs. 1, 2, 3 and 4b. (DOCX 17 kb)

Additional file 2: **Table S2.** Number of examined mice in Fig. 4d. (DOCX 17 kb)

Additional file 3: **Table S3.** Antibodies, working dilutions, and methods for antigen retrieval. (DOCX 18 kb)

## Data Availability

The datasets used and analyzed during the current study available from the corresponding author on reasonable request.

## Abbreviations

AB pH 2.5: Alcian blue pH 2.5
AhR: Aryl-hydrocarbon receptor
B6-CA-AhR: C57BL/6-background CA-AhR
BrdU: Bromodeoxyulidine
CA-AhR: Constitutively-active AhR
CDX2: Caudal type homeobox 2
CYP1A1: Cytochrome P450, family 1, subfamily a, polypeptide 1
EM cyst: Extramucosal cyst
FICZ: 6-formylindolo[3,2-b]carbazole
FVB-CA-AhR: FVB/N-background CA-AhR
GCP: Gastritis cystica profundal
GSII: *Griffonia simplicifolia* lectin II
HE: Hematoxylin-eosin
IM cyst: Intramucosal cyst
PAS: Periodic acid Schiff
PBS: Phosphate-buffered saline
PCNA: Proliferating cell nuclear antigen
SEM: Scanning electron microscopy
SPEM: Spasmolytic polypeptide-expressing metaplasia
SV40: Simian virus 40
TCDD: 2,3,7,8-tetracholorodibenzo-p-dioxin
TFF2: Trefoil factor 2
wks: weeks
