# Supplementary material for: Histopathological features of the proper gastric glands in FVB/N-background mice carrying constitutively-active aryl-hydrocarbon receptor
Source: BMC Gastroenterol. 2019 Jun 21;19:102. doi: 10.1186/s12876-019-1009-x (PMC6588904; doi:10.1186/s12876-019-1009-x)
Supplement: Supplementary file 1 — Table S1. Number of examined mice in Figs. 1, 2, 3 and 4b. (DOCX 17 kb) [file 12876_2019_1009_MOESM1_ESM.docx]

| **Table S1.** Number of examined mice in Fig.1, 2, 3 and 4b. | | | | |
| --- | --- | --- | --- | --- |
|  | FVB-CA-AhR | | wild-type | |
| age(weeks) | male | female | male | female |
| 6 | 5 | - | 4 | - |
| 7 | 4 | - | - | - |
| 9 | 5 | 5 | 4 | 5 |
| 13 | 6 | 5 | 4 | 4 |
| 16 | 7 | 6 | 5 | 4 |
| 28 | 5 | 5 | 4 | 4 |
| 33 | - | 5 | - | - |
