# Supplementary material for: Histopathological features of the proper gastric glands in FVB/N-background mice carrying constitutively-active aryl-hydrocarbon receptor
Source: BMC Gastroenterol. 2019 Jun 21;19:102. doi: 10.1186/s12876-019-1009-x (PMC6588904; doi:10.1186/s12876-019-1009-x)
Supplement: Supplementary file 2 — Table S2. Number of examined mice in Fig. 4d. (DOCX 17 kb) [file 12876_2019_1009_MOESM2_ESM.docx]

| **Table S2.** Number of examined mice in Fig. 4d. | | | | |
| --- | --- | --- | --- | --- |
|  | FVB-CA-AhR | | wild-type | |
| age(weeks) | male | female | male | female |
| 9 | 5 | 5 | 4 | 4 |
| 13 | 6 | 5 | 4 | 4 |
| 16 | 5 | 5 | 5 | 4 |
| 28 | 5 | 5 | 4 | 4 |
