# Supplementary material for: Histopathological features of the proper gastric glands in FVB/N-background mice carrying constitutively-active aryl-hydrocarbon receptor
Source: BMC Gastroenterol. 2019 Jun 21;19:102. doi: 10.1186/s12876-019-1009-x (PMC6588904; doi:10.1186/s12876-019-1009-x)
Supplement: Supplementary file 3 — Table S3. Antibodies, working dilutions, and methods for antigen retrieval. (DOCX 18 kb) [file 12876_2019_1009_MOESM3_ESM.docx]

| **Table S3**. Antibodies, working dilutions, and methods for antigen retrieval. | | | | |
| --- | --- | --- | --- | --- |
| Antibody | Source | Dilution | Antigen retrieval | Heating condition |
| Goat anti-CYP1A1 | Santa Cruz Biotechnology, Texas, USA | 1:300 | 10 mM Citrate buffer (pH 6.0) | 105℃, 20 min |
| Goat anti-PCNA | Santa Cruz Biotechnology, Texas, USA | 1:2000 | 10 mM Citrate buffer (pH 6.0) | 105℃, 20 min |
| Rat anti-BrdU | abcam, Tokyo, Japan | 1:200 | 10 mM Citrate buffer (pH 6.0) | 105℃, 20 min |
| Rat anti-B220 | Cedarlane, Ontario, Canada | 1:1600 | 0.1 % pepsin/0.2 N HCl | 37℃, 5 min |
| Rabit anti-CD3 | Nichirei, Tokyo, Japan | 1:200 | 20 mM Tris–HCl (pH 9.0) | 105℃, 20 min |
| Lectins | Vector Laboratories, Inc. Burlingame, CA, USA | 1:100 | not conducted | − |
| Mouse anti-CDX2 | BioGenex, Hague, Netherlands | 1:1 | 20 mM Tris–HCl (pH 9.0) | 105℃, 20 min |
| Rabbit anti-TFF2 | Proteintech, Rosemont, IL 60018, USA | 1:3200 | 20 mM Tris–HCl (pH 9.0) | 105℃, 20 min |
| Rabbit anti-pepsinogen | Proteintech, Rosemont, IL 60018, USA | 1:100 | - | - |
| CYP1A1: cytochrome P450, family 1, subfamily a, polypeptide 1 | | | | |
| PCNA: proliferating cell nuclear antigen | | | | |
| BrdU: bromodeoxyulidine | | | | |
| CDX2: caudal type homeobox 2 | | | | |
| TFF2: trefoil factor 2 | | | | |
